# Evolutionary analysis of foot-and-mouth disease virus serotype SAT 1 isolates from east africa suggests two independent introductions from southern africa

**DOI:** 10.1186/1471-2148-10-371

**Published:** 2010-11-30

**Authors:** Abraham K Sangula, Graham J Belsham, Vincent B Muwanika, Rasmus Heller, Sheila N Balinda, Charles Masembe, Hans R Siegismund

**Affiliations:** 1 Makerere University, Institute of Environment and Natural Resources, Molecular Biology Laboratory, P. O. Box 7298, Kampala, Uganda; 2 National Veterinary Institute, Technical University of Denmark, Lindholm, DK-4771 Kalvehave, Denmark; 3 Department of Biology, University of Copenhagen, Ole Maaløes Vej 5, DK-2200, Copenhagen N, Denmark; 4 Foot-and-Mouth Disease Laboratory, Embakasi, P. O. Box 18021, 00500, Nairobi, Kenya

## Abstract

**Background:**

In East Africa, foot-and-mouth disease virus serotype SAT 1 is responsible for occasional severe outbreaks in livestock and is known to be maintained within the buffalo populations. Little is known about the evolutionary forces underlying its epidemiology in the region. To enhance our appreciation of the epidemiological status of serotype SAT 1 virus in the region, we inferred its evolutionary and phylogeographic history by means of genealogy-based coalescent methods using 53 VP1 coding sequences covering a sampling period from 1948-2007.

**Results:**

The VP1 coding sequence of 11 serotype SAT 1 FMD viruses from East Africa has been determined and compared with known sequences derived from other SAT 1 viruses from sub-Saharan Africa. Purifying (negative) selection and low substitution rates characterized the SAT 1 virus isolates in East Africa. Two virus groups with probable independent introductions from southern Africa were identified from a maximum clade credibility tree. One group was exclusive to Uganda while the other was present within Kenya and Tanzania.

**Conclusions:**

Our results provide a baseline characterization of the inter-regional spread of SAT 1 in sub-Saharan Africa and highlight the importance of a regional approach to trans-boundary animal disease control in order to monitor circulating strains and apply appropriate vaccines.

## Background

Foot-and-mouth disease (FMD) is an acute, highly communicable and economically important disease of livestock and it also affects wild ruminants [1]. The causative agent, foot-and-mouth disease virus (FMDV) belongs to the *Aphthovirus *genus in the family *Picornaviridae*. Its positive-sense, single-stranded RNA genome of 8.5 kb is translated into a polyprotein which is post-translationally cleaved to 4 structural (VP1, VP2, VP3, VP4) and 8 nonstructural proteins [2]. The structural proteins form the capsid of the virion and, with the exception of VP4, are surface exposed. The VP1 is involved in the interaction with the host cells via the RGD-dependent integrins [3]. The coding sequence for VP1 has been widely used in studies of evolutionary dynamics of FMDV needed for the understanding of the epidemiological patterns of these viruses and for determining possible sources of outbreaks [4-6]. The genetic diversity of FMDV is a consequence of the high mutation rate due to the error-prone RNA polymerase lacking proofreading activity [7].

There are seven immunologically distinct serotypes (O, A, C, SAT 1, SAT 2, SAT 3 and Asia 1) of FMDV, each with a wide spectrum of antigenic and epidemiological subtypes distributed around the world [5]. The Southern Africa Territories (SAT) serotypes are restricted in their distribution mainly to sub-Saharan Africa and they co-exist with the Euro-Asiatic (O, A, C) serotypes in the East African region although serotype C has not been reported since 2004. In southern Africa, the epidemiology of the SAT serotypes is mainly associated with African buffalos (*Syncerus caffer*) which act as reservoirs and sources of outbreaks [8,9]. In eastern Africa, FMD is prevalent in wildlife and within the African buffalo in particular although their role in the epidemiology of the disease has not been as widely studied as in southern Africa. Most outbreaks of FMD in the region are reported among livestock populations. The African buffalo has been reported to be a carrier of the SAT serotypes but not the Euro-Asiatic serotypes in East Africa [10-12]. This is similar to the situation in southern Africa. Widespread animal movements in the eastern Africa region are possibly responsible for long-term circulation and reintroductions of FMDV strains, including SAT 1 [13]. However, little quantitative information exists about the extent of such livestock and wildlife mediated dispersal of FMDV as well as the origin and evolutionary history of the SAT 1 viruses circulating in eastern Africa [13,14]. Furthermore, the connectivity between the individual countries and the main routes of dispersal remain unknown, although such information would be of great value in containing the spread of the disease and avoiding introduction of novel strains against which existing vaccine programs may offer little protection.

We have investigated the emergence of FMDV SAT 1 diversity in the region by inferring the phylogeographic history by means of genealogy-based coalescent methods. Furthermore, we have tested for evidence of recombination in the data set which is known to bias phylogenetic inferences as described previously [15-17].

## Results

### Phylogenetic relationships, substitution rates and divergence times

**Figure 1:**
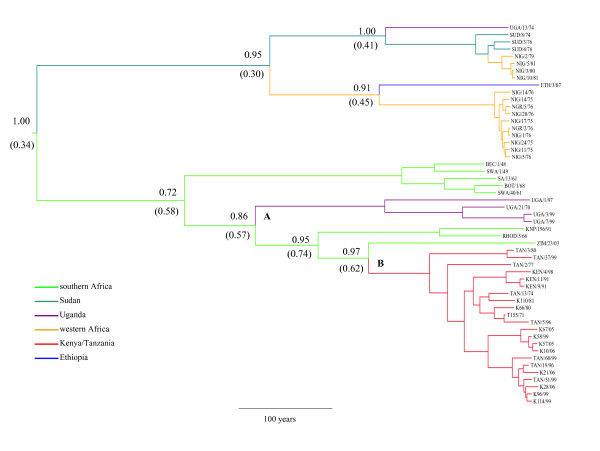
**Bayesian time-scaled phylogeny of FMDV serotype SAT 1 with inferred geographical location states**. Maximum clade credibility tree of SAT 1 viruses based on complete VP1 coding sequences inferred using BEAST assuming a constant size coalescent prior showing lineage divergence since the most recent common ancestor. The inferred geographical location of each tree node is marked by a colour code defined in the insert legend. The posterior clade probabilities as well as the posterior geographical location state (in parenthesis) are shown for selected tree nodes. East African lineages A and B are marked.

The VP1 coding sequences of 11 additional serotype SAT 1 FMD viruses from East Africa have been determined. Using this information, the complete VP1 coding sequences of 8 southern Africa, 14 western Africa, 3 Sudanese, 1 Ethiopian and 27 East Africa FMD serotype SAT 1 viruses from the period 1948 to 2007 were analysed to determine phylogenetic relationships, phylogeography, divergence times and substitution rates. Dating of the common root of the samples showed considerable uncertainty in determination with a mean estimate for the most recent common ancestor (TMRCA) at 538 years before present (ybp) (95% highest posterior density (HPD): 228-897 ybp). The inferred maximum clade credibility (MCC) tree is shown in Figure 1 with the posterior probabilities for the branches shown. The East African SAT 1 viruses formed two main clades (lineages) labelled A and B supported by high posterior probabilities. The Ugandan viruses differed from those of Tanzania and Kenya and are of mainly one lineage (A) while one isolate (UGA/13/74) grouped with viruses of the Sudan and western Africa. Kenyan and Tanzanian viruses grouped together in lineage B and were related to a Zimbabwean isolate. Only little geographic structure was observed within lineage B isolates from Kenya and Tanzania, suggesting high migration rates between these countries. The mean nt substitution rate was 1.30 × 10^-3 ^substitutions/site/year (s/s/yr) (95% HPD: 5.43 × 10^-4^-2.18 × 10^-3^) with distinct variation in rates among the clades. We analysed the East African viruses (comprising 27 samples) separately in BEAST and found relatively lower rates at 2.75 × 10^-4 ^s/s/yr (4.69 × 10^-5^-7.39 × 10^-4^), while the western African viruses (comprising 14 samples) had higher rates at 6.91 × 10^-3^s/s/yr (3.32 × 10^-3^-1.04 × 10^-2^).

While the location of the root of the SAT1 tree could not be identified with particular confidence (Bayes factor, BF = 1.5 comparing the posterior probability of the root being in southern Africa against Sudan), there was relatively strong support for the location of several of the remaining nodes in the MCC tree (Figure 1). From the location-annotated MCC tree, two separate introductions from southern Africa to East Africa were supported by the data, namely one leading to lineage A and one leading to lineage B. In addition, there was strong support for two separate introductions of SAT 1 from Sudan to western Africa. Bayes factor tests revealed that the most significant routes of inter-regional dispersal were the Sudan-western Africa (BF = 21.4) and southern Africa-Kenya/Tanzania (BF = 4.5). No link between the Ugandan and the Kenyan/Tanzanian samples (between lineage A and B) could be identified, and this was in fact found to be the link with the second-lowest posterior support. The western Africa-Kenya/Tanzania link had the lowest support (results not shown).

### Predominant purifying selection in the VP1 coding region of FMDV SAT 1

The majority of codons in the VP1 coding region of FMDV SAT 1 appeared to be under purifying (negative) selection. Of the 221 codons analysed, 153 were found to be under negative selection using three methods (single-likelihood ancestor counting, SLAC, at *P *= 0.1, fixed effects likelihood, FEL, at *P *= 0.1 and random effects likelihood, REL, with BF > 50) as summarized in Table 1. Five sites (codons 47, 61, 99, 143 and 147) were identified to be under positive selection by at least one method but no site was identified by all the three methods together at values of *P *= 0.1 (SLAC and FEL) or BF > 50 (REL). However, at *P *= 0.2, codon 147 (H/N/E/T, 2 codon positions before the receptor binding motif RGD) was identified by all the methods and was mostly likely to be under true positive selection.

**Table 1:** Evidence for negative and positive selection using SLAC, FEL, and REL methods

Number of sites	SLAC (*P *< 0.1)	FEL (*P *< 0.1)	REL (BF > 50)	Integrated
Positively selected	0	2	4	5
Negatively selected	143	152	112	153

A summary of the number of codon sites identified by the various methods to have been under selection at the default significance values for the three methods.

The genetic algorithm (GA) branch analysis showed that 5 rate classes were supported with a large number of models (over 1500 in the 95% confidence set). No branches had significant support for *dN *>*dS *although differences existed in the branch selection pattern indicating that some branches may have been under weak positive selection.

### Recombination

The genetic algorithm for recombination detection (GARD) detected a putative recombination breakpoint at nucleotide position 168 with a change in Akaike's Information Criterion (AIC_c_) of more than100 which suggested support for the recombination model while the Kishino-Hasegewa test showed support for significant topological incongruence at *P *= 0.01. Indeed, the exploratory analysis using the recombination detection programme (RDP2) had at least one method detect some recombinant sequences (TAN/60/99 and K66/80). However, further analysis did not support the view that these sequences were recombinant and the exclusion of these sequences from the analysis did not affect the phylogenetic results, indicating that they are not likely to be true recombinants (results not shown).

## Discussion

FMDV serotype SAT 1 virus strains from East Africa analysed in this study grouped into 2 distinct clades (lineages with > 20% nucleotide divergence) designated here as lineages A and B. While one of these lineages (A) was found exclusively in Uganda, the other had virus strains from Tanzania and Kenya. Over the whole sampling period, Kenyan and Tanzanian isolates were interspersed in one clade of the phylogenetic tree, suggesting that these countries form a single ecosystem for SAT 1. The separate introduction of lineages A and B to eastern Africa from southern Africa was supported by the high posterior probabilities of the location states in the phylogeographic analysis. A close association of Kenyan/Tanzanian and southern African lineages has been observed earlier [13], but the link between Ugandan and southern Africa lineages reported here reveals a previously undiscovered aspect of the ancestry of the East African SAT 1 lineages. This new finding was not due to a different data set, but rather to our Bayesian phylogeographic analysis framework. When we constructed a neighbour-joining tree using similar methods to [13] using our data set, we were not able to infer a southern African origin for the Ugandan lineage.

Several interesting aspects about the history of sub-Saharan SAT 1 viruses emerge from our continental phylogeographic approach. First, we found that the most likely root location of SAT 1 is in southern Africa. Because of the relatively deep root of the tree (~538 ybp), we could not achieve unequivocal posterior support for this root location (also see [18]). We found a strong link between western African and Sudanese SAT 1 sequences (in agreement with [13]) and our results suggest that the route of entry of SAT 1 into western Africa has been along the Sahel rather than through the rain forest belt surrounding equatorial Africa. A Ugandan isolate from 1974 was found to belong to a lineage otherwise consisting of Sudanese, Ethiopian and western African strains, and the phylogeographic analysis suggested this was an incursion from Sudan. Hence, Ugandan SAT 1 strains appear to be derived from two different sources, southern Africa and Sudan, respectively.

The sampling scheme used in this study may to some extent have affected the outcome of the phylogeographic analysis. For example, we cannot exclude that the inclusion of more samples from Uganda would alter the posterior state probability of some nodes in the tree to reflect an earlier introduction of SAT 1 into Uganda. Given that Uganda is represented in both of the two major clades, it may have played a more prominent role connecting southern African SAT 1 viruses with those of Sudan, Ethiopia and western Africa. Such a scenario seems plausible given the central location of Uganda according to our definition of location states. Furthermore, we cannot exclude that additional samples from Uganda will show phylogenetic affinity with the surrounding countries. This could be tested by acquiring more Ugandan samples. In fact, more recent SAT 1 virus isolates from Uganda have grouped within the Ugandan lineage A [19], in agreement with the phylogeographic conclusions reported here. In general, however, we stress that our findings should be viewed as a null hypothesis about continental SAT 1 dispersal against which studies based on more comprehensive sampling can be tested. Denser sampling (both temporally and spatially) can be expected to reveal novel dispersal patterns not observed here and further address the fine-scale historical movement of the serotype.

The substitution rate inferred in our study differs considerably from [20]. This leads to a significantly deeper tree, and hence it is difficult for us to put our results into a historical context that includes all FMDV serotypes. Our mean estimate of 538 ybp for the TMRCA of SAT 1 actually predates that of the whole FMDV found in [20], although it is within the 95% HPDs reported in that study (218-1250 ybp). We caution that the time line of our phylogeographic tree should not be regarded as conclusive and that further studies are needed to establish the rate of evolution in FMDV. Our inferred rate is, however, closer to the reported mean rate of evolution across all serotypes (2.48 × 10^-3^) for the VP1 coding sequence [20]. In [20], the SAT 1 virus sequence was found to have a roughly 3-fold faster rate than the species average. We speculate that this exceptionally fast rate could be derived from the sampling scheme in [20], where many of the included SAT 1 isolates are from the same epidemic outbreaks. This tends to yield faster rates of evolution, since what is recovered is actually the mutation rate rather than the long-term substitution rate subject to selection and other forces [21], leading to a bias towards higher rates and a shallower tree. In accordance with this, we did find much faster rates of evolution in the western African samples; all collected during two epidemic outbreaks each spanning just two years. However, regionally variable evolutionary rates may in fact reflect real differences in the epidemiological dynamics and host-interaction of FMDV. For example, buffalos and other wildlife may play a more prominent role in the epidemiology of SAT 1 in eastern than in western Africa, and this may give rise to changed patterns of evolution of virus lineages in the two regions. Considerable localized differentiation in evolutionary rates has not previously been observed in FMDV, and although potentially informative concerning epidemiology and evolution, it also complicates evolutionary estimates based on global or widespread sample collections. Given these two (not necessarily mutually exclusive) causes of the observed rate heterogeneity, it is vital that future studies address the caveats in using the VP1 coding sequence to infer evolutionary rates and history.

Purifying (negative) selection was the most predominant evolutionary force at play among the SAT 1 viruses. At least 153 codon positions including the RGD motif (amino acid residue positions 149-151) of VP1, required for receptor interaction, were estimated to be under purifying selection signifying amino acid conservation as reflected in the low evolutionary rates. There was less evidence for positive selection although a few sites may have been under adaptive selection. Amino acid sites that are distinct between the regional virus groups as well as conservation of the RGD motif were observed when inferred using MEGA version 4 [22] and is in agreement with previous reports [13]. These evolutionary patterns may reflect the observed apparent long term circulation of some virus strains in the region previously reported in [13]. It has also been observed that genetic heterogeneity may be limited by evolutionary constraints [23]. There was no evidence for the presence of recombination within the VP1 coding sequences (in agreement with observations that recombination is largely restricted to non-structural coding regions with very few phylogenetic incongruities in the capsid proteins [24-26]) adding confidence to our results.

## Conclusions

We have inferred the most likely phylogeographic history of SAT 1 in sub-Saharan Africa. We found evidence that the SAT 1 viruses circulating in Uganda and Kenya/Tanzania represent independent phylogeographic lineages. Kenya and Tanzania appear to experience a much greater exchange of viruses at their respective southern and northern borders through the trans-boundary livestock and wildlife movements (a common feature in this area) than with Uganda. This highlights the importance of a regional approach to trans-boundary animal disease control. It is apparent from the SAT 1 analysis presented here that monitoring of the emerging strains in the region is required for the success of vaccination strategies.

## Methods

### Virus Isolates

Eleven (10 Kenyan and 1 Tanzanian) SAT 1 virus isolates for this study (collected between 1977 and 2006) were obtained from the Embakasi FMD laboratory in Nairobi which is a repository of all FMD sample materials collected in Kenya. Virus was isolated from clinical material according to standard procedures on baby hamster kidney (BHK) cells. The details of the isolates are shown in Table 2.

**Table 2:** List of the SAT 1 virus isolates included in this study

Isolate Code^a^	District/Country	Geographical group^b^	Accession No.
BEC/1/48	Bechuanaland (Botswana)	SA	AY593838
SWA/1/49	South West Africa (Namibia)	SA	AY593840
SA/13/61	South Africa	SA	AY593842
SWA/40/61	South West Africa (Namibia)	SA	AY593843
RHO/5/66	Rhodesia (Zimbabwe)	SA	AY593846
BOT/1/68	Botswana	SA	AY593845
UGA/21/70	Uganda	UG	WRL^c^
T155/71	Tanzania	KT	HQ267519^e^
SUD/8/74	Sudan	SU	AY441998
TAN/13/74	Tanzania	KT	AY442001
UGA/13/74	Uganda	UG	AY442010
NIG/11/75	Nigeria	WA	AF431711
NIG/14/75	Nigeria	WA	AF431709
NIG/17/75	Nigeria	WA	AF431712
NIG/24/75	Nigeria	WA	AF431714
NIG/1/76	Nigeria	WA	AF431721
NIG/5/76	Nigeria	WA	AF431723
NIG/14/76	Nigeria	WA	AF431725
NIG/20/76	Nigeria	WA	AF431727
NGR/2/76	Niger	WA	AF431718
NGR/5/76	Niger	WA	AF431720
SUD/3/76	Sudan	SU	AY441966
SUD/4/76	Sudan	SU	AY441997
TAN/2/77	Tanzania	KT	AY442008
NIG/2/79	Nigeria	WA	AF431728
K66/80	Narok, Kenya	KT	HQ267520^e^
NIG/3/80	Nigeria	WA	AF431729
TAN/3/80	Tanzania	KT	AY442006
K110/81	Kiambu, Kenya	KT	HQ267521^e^
NIG/5/81	Nigeria	WA	AF431730
NIG/10/81	Nigeria	WA	AF431731
KEN/9/91	Kenya	KT	AY441995
KEN/11/91	Kenya	KT	AY441994
KNP/196/91^d^	South Africa	SA	AF283429
TAN/5/96	Tanzania	KT	AY442007
TAN/19/96	Tanzania	KT	AY442013
UGA/1/97^d^	Uganda	UG	AF283439
KEN/4/98	Kenya	KT	AY441993
K58/99	Thika, Kenya	KT	HQ267522^e^
K96/99	Kajiado, Kenya	KT	HQ267523^e^
K114/99	Nairobi, Kenya	KT	HQ267524^e^
TAN/37/99	Tanzania	KT	AY442005
TAN/51/99	Tanzania	KT	AY442004
TAN/60/99	Tanzania	KT	AY442002
UGA/3/99	Uganda	UG	AY442009
UGA/7/99	Uganda	UG	AY442011
ZIM/23/03	Zimbabwe	SA	WRL^c^
K57/05	Thika, Kenya	KT	HQ267525^e^
K67/05	Trans Nzoia, Kenya	KT	HQ267526^e^
K10/06	Thika, Kenya	KT	HQ267527^e^
K21/06	Nyeri, Kenya	KT	HQ267528^e^
K28/06	Keiyo, Kenya	KT	HQ267529^e^
ETH/3/07	Ethiopia	ET	EJ798154

^a ^The letters indicate the country of origin of the isolate while the last numerical field refers to the year of isolation. ^b ^Geographical group assignment according to the six defined regions of sub-Saharan Africa: SA (southern Africa), UG (Uganda), KT (Kenya/Tanzania), SU (Sudan), ET (Ethiopia) WA (western Africa). ^c ^WRL = From World reference laboratory sequence data website http://www.wrlfmd.org/fmd_genotyping/prototypes.htm. ^d ^African buffalo origin. ^e^Sequence generated in this study.

### Viral RNA extraction, cDNA synthesis and amplification

Total RNA was extracted and cDNA synthesized as previously described [27]. The complete VP1 coding region was amplified using the primer pair, FMD AKS (5'-ATGGGACACAGGTCTGAACTCGA-3') and FMD-2B58 [28] applying PCR reagent volumes and conditions as previously described [27]. PCR products were visualized, purified and cycle-sequenced using the same primers as for PCR above.

### Phylogeographic analysis

In addition to the eleven sequences generated in the study, 42 (17 from East Africa and 25 the rest of Africa) other complete VP1 coding sequences available in the GenBank covering a sampling period from 1948-2007 were included to put the results from East Africa into a continental SAT 1 context.

The sequences were assembled and aligned using the software program Geneious version 4.6 [29]. The best fitting nucleotide substitution model was tested by means of a hierarchical likelihood ratio test (LRT) and the Akaike information criteria (AIC) as implemented in MrModeltest version 2.2 software [30] and executed in PAUP* version 4b10 software [31]. The selected model was general time-reversible (GTR) [32] with gamma-distributed rates among sites and a proportion of invariable sites.

Phylogenetic relationships, evolutionary rates and population size changes were co-estimated for the whole data set and geographic subsets using a Bayesian Markov Chain Monte Carlo (MCMC) method implemented in the BEAST (Bayesian evolutionary analysis sampling trees) software version 1.6.0 package [33]http://beast.bio.ed.ac.uk using the selected model of nucleotide substitution. The method utilizes the sampling time of the sequences to infer rates of evolution along lineages, time of TMRCA and demographic history. A recent extension of the software allows tracking of the geographic location state along the phylogenetic tree, yielding posterior estimates of the location of each branch/node in the tree given the phylogenetic uncertainties [18]. Given our limited data set with low representation of many countries, we defined the geographical states as six coherent regions roughly corresponding to areas separated by known topotype boundaries. The regions include: western Africa, Ethiopia, Sudan, Uganda, Kenya/Tanzania and southern Africa (Table 2). We used a Bayesian stochastic search variable selection (BSSVS) without distance informed priors on diffusion rates, as this has been shown not to improve confidence in the phylogeographical state assignment when dispersal patterns are complex such as with many viruses (e.g. [18]). Rate indicator log files were inspected in Tracer software version 1.4 http://tree.bio.ed.ac.uk/software/tracer/, and Bayes factor tests were carried out to test the most significant routes of dispersal using the Rate Indicator BF tool of the BEAST package.

In a preliminary analysis, we tested four different demographic models/coalescent priors as suggested in [33]. In addition, we tested the appropriateness of a strict clock versus various versions of relaxed clocks available in BEAST [34]. This process of model selection suggested the constant population size model with an uncorrelated exponential clock to be the best fit to the data. We used a HKY+G+I substitution model [35] with four rate categories, as in a recent influenza study ([18], Additional file 1). The MCMC chains were run long enough (100 million steps) to allow high effective sample sizes (ESSs) (above 250 for most parameters, minimum 100 for all parameters) with a 10% burn-in as viewed in Tracer. Statistical uncertainties of the substitution rates and the MRCA were summarized as the lower 95%, mean, and upper 95% values of the HPD interval. Mean evolutionary rates (averaged over branches weighted by their lengths) were measured as the number of nucleotide substitution per site per year (s/s/y). Maximum clade credibility trees were obtained using Tree Annotator program in BEAST and visualized with FigTree version 1.1.2 software http://tree.bio.ed.ac.uk/software/figtree/.

### Selection and recombination detection

Tests for selection were performed using four methods which estimate selection in a phylogenetic context available in the Datamonkey web interface [36]. The best-fitting nucleotide substitution model was selected using the automated link. To identify codon sites under positive (adaptive) or negative (purifying) selection, we used the single-likelihood ancestor counting, the fixed effects likelihood and the random effects likelihood methods. The SLAC and FEL methods estimate selection on a site-by-site basis with the former method comparing observed to expected synonymous and non-synonymous rates while the latter uses two models which assume independent and equal rates and a likelihood ratio test to determine significance. The REL method determines independent general discrete distributions for the global synonymous and non-synonymous rates using a codon based model which are then used as priors for Empirical Bayes analysis of site selection [36]. The integrative selection analysis option in Datamonkey was then used to increase confidence on the estimation of selection at a site if all three methods support it. To test the hypothesis that different selective environments were acting on the branches of the phylogeny, we used the GA branch method to estimate *dN*/*dS*.

To add confidence to our coalescent inferences, the presence of recombination in the data was tested using the GARD method [37] on the Datamonkey server with topological incongruence significance estimated by the Kishino-Hasegawa test [38] and also by the exploratory methods implemented in RDP version 2 beta 0.8 software [39] which included; RDP, [40] GENECONV, [41] Bootscan, [42] MaxChi, [43] and Chimaera [44].

## Authors' contributions

AKS, GJB, VM and HRS designed and conceived the study. AKS, SNB and CM generated, collected and aligned the sequences. AKS, RH, SNB and HRS carried out the analysis of the data. AKS, GJB, RH, VM and HRS wrote the paper. All authors read and approved the final manuscript.

## Supplementary Material

Additional file 1 **BEAST XML file for the FMDV serotype SAT 1 analysis**. Input XML file used for BEAST relaxed molecular clock and constant size prior analysis of VP1 coding region. Click here for file

## References

[B1] Grubman MJ Baxt B Foot-and-mouth disease Clinical Microbiology Reviews 2004 17 2 465 493 10.1128/CMR.17.2.465-493.2004 15084510 PMC387408

[B2] Belsham GJ Distinctive features of foot-and-mouth disease virus, a member of the picornavirus family; aspects of virus protein synthesis, protein processing and structure Progress in Biophysics and Molecular Biology 1993 60 241 260 10.1016/0079-6107(93)90016-D 8396787 PMC7173301

[B3] Jackson T King AMQ Stuart DI Fry E Structure and receptor binding Virus Research 2003 91 33 46 10.1016/S0168-1702(02)00258-7 12527436

[B4] Haydon DT Samuel AR Knowles NJ The generation and persistence of genetic variation in foot-and-mouth disease virus Preventive Veterinary Medicine 2001 51 111 124 10.1016/S0167-5877(01)00210-0 11530198

[B5] Knowles NJ Samuel AR Molecular epidemiology of foot-and-mouth disease virus Virus Research 2003 91 65 80 10.1016/S0168-1702(02)00260-5 12527438

[B6] Tully DC Fares MA Unravelling selection shifts among foot-and-mouth disease virus (FMDV) serotypes Evolutionary Bioinformatics 2006 2 211 225 PMC2674665 19455214

[B7] Domingo E Escarmí's C Baranowski E Ruiz-Jarabo CM Carrillo E Núñez JI Sobrino F Evolution of foot-and-mouth disease virus Virus Research 2003 91 47 63 10.1016/S0168-1702(02)00259-9 12527437

[B8] Condy JB Hedger RS Hamblin C Barnett ITR The duration of foot-and-mouth disease virus carrier state in African buffalo (i) in the individual animal and (ii) in a free-living herd Comparative Immunology, Microbiology and Infectious Diseases 1985 8 3/4 259 265 10.1016/0147-9571(85)90004-9 3004803

[B9] Thomson GR Vosloo W Bastos ADS Foot and mouth disease in wildlife Virus Research 2003 91 145 161 10.1016/S0168-1702(02)00263-0 12527441

[B10] Anderson EC Doughty WJ Anderson J Paling R The pathogenesis of foot-and-mouth disease in the African buffalo (*Syncerus caffer*) and the role of this species in the epidemiology of the disease in Kenya Journal of Comparative Pathology 1979 89 511 519 10.1016/0021-9975(79)90045-8 232107

[B11] Ayebazibwe C Mwiine FN Balinda SN Tjørnehøj K Masembe C Muwanika VB Okurut ARA Siegismund HR Alexandersen S Antibodies against foot-and-mouth disease (FMD) virus in African buffalos (*Syncerus caffer*) in selected national parks in Uganda (2001-2003) Transboundary and Emerging Diseases 2010 57 286 292 20561289 10.1111/j.1865-1682.2010.01147.x

[B12] Bronsvoort BMDC Parida S Handel I McFarland S Fleming L Hamblin P Kock R Serological survey for foot-and-mouth disease virus in wildlife in eastern Africa and estimation of test parameters of a nonstructural protein enzyme-linked immunosorbent assay for buffalo Clinical and Vaccine Immunology 2008 15 6 1003 1011 10.1128/CVI.00409-07 18385460 PMC2446625

[B13] Sahle M Dwarka RM Venter EH Vosloo W Comparison of SAT-1 foot-and-mouth disease virus isolates obtained from East Africa between 1971 and 2000 with viruses from the rest of sub-Saharan Africa Archives of Virology 2007 152 797 804 10.1007/s00705-006-0893-x 17187294

[B14] Bastos ADS Haydon DT Forsberg R Knowles NJ Anderson EC Bengis RG Nel LH Thomson GR Genetic heterogeneity of SAT-1 type foot-and-mouth disease viruses in southern Africa Archives of Virology 2001 146 1537 1551 10.1007/s007050170077 11676416

[B15] Schierup MH Hein J Consequences of recombination on traditional phylogenetic analysis Genetics 2000 156 879 891 11014833 10.1093/genetics/156.2.879 PMC1461297

[B16] Moya A Holmes EC González-Candelas F The population genetics and evolutionary epidemiology of RNA viruses Nature Reviews | Microbiology 2004 2 279 288 10.1038/nrmicro863 PMC7096949 15031727

[B17] Heath L van der Walt E Varsani A Martin DP Recombination patterns in aphthoviruses mirror those found in other picornaviruses Journal of Virology 2006 80 23 11827 11832 10.1128/JVI.01100-06 16971423 PMC1642601

[B18] Lemey P Rambaut A Drummond AJ Suchard MA Bayesian phylogeny finds its roots PLoS Computational Biology 2009 5 9 1 16 10.1371/journal.pcbi.1000520 PMC2740835 19779555

[B19] Ayebazibwe C Mwine FN Tjørnehøj K Balinda SN Muwanika VB Okurut AAR Belsham GJ Normann P Siegismund HR Alexandersen S The role of African buffalos (*Syncerus caffer*) in the maintenance of foot-and-mouth disease in Uganda BioMed Central, Veterinary Research in press 10.1186/1746-6148-6-54 PMC3022570 21143994

[B20] Tully DC Fares MA The tale of a modern animal plague: tracing the evolutionary history and determining the time-scale for foot and mouth disease virus Virology 2008 382 250 256 10.1016/j.virol.2008.09.011 18945462

[B21] Ho SYW Phillips MJ Cooper A Drummond AJ Time dependency of molecular rate estimates and systematic overestimation of recent divergence times Molecular Biology and Evolution 2005 22 7 1561-1568 10.1093/molbev/msi145 15814826

[B22] Tamura K Dudley J Nei M Kumar S MEGA4: Molecular evolutionary genetics analysis (MEGA) software version 4.0 Molecular Biology and Evolution 2007 24 8 1596 1599 10.1093/molbev/msm092 17488738

[B23] Haydon DT Bastos AD Knowles NJ Samuel AR Evidence for positive selection in foot-and-mouth disease virus capsid genes from field isolates Genetics 2001 157 7 15 11139487 10.1093/genetics/157.1.7 PMC1461471

[B24] Carrillo C Tulman ER Delhon G Lu Z Carreno A Vagnozzi A Kutish GF Rock DL Comparative genomics of foot-and-mouth disease virus Journal of Virology 2005 79 10 6487 6504 10.1128/JVI.79.10.6487-6504.2005 15858032 PMC1091679

[B25] Tully DC Fares MA Shifts in the selection-drift balance drive the evolution and epidemiology of foot-and-mouth disease virus Journal of Virology 2009 83 2 781 790 10.1128/JVI.01500-08 19004952 PMC2612391

[B26] Jackson AL O'Neill H Maree F Blignaut B Carrillo C Rodriguez L Haydon DT Mosaic structure of foot-and-mouth disease virus genomes Journal of General Virology 2007 88 487 492 10.1099/vir.0.82555-0 17251567

[B27] Sangula AK Siegismund HR Belsham GJ Balinda SN Masembe C Muwanika VB Low diversity of foot-and-mouth disease serotype C virus in Kenya: evidence for probable vaccine strain re-introductions in the field Epidemiology and Infection 2010 Available on Cambridge Journals Online, 25 March 2010 10.1017/S0950268810000580 20334728

[B28] Knowles NJ Samuel AR Polymerase chain reaction amplification and cycle sequencing of the 1 D gene of foot-and-mouth disease viruses Session of the research group of the standing technical committee of the European commission for the control of foot-and-mouth disease. 19-22 September 1994 1995 Vienna, Austria: FAO, Rome

[B29] Drummond AJ Ashton B Cheung M Heled J Kearse M Moir R Stones-Havas S Thierer T Wilson A Geneious v4.6 2009 http://www.geneious.com/

[B30] Nylander JAA MrModeltest v2 Program distributed by the author 2004 Evolutionary Biology Centre, Uppsala University

[B31] Swofford DL PAUP*. Phylogenetic analysis using parsimony (*and other methods). Version4 2003 4 Sunderland, Massachusetts: Sinauer Associates

[B32] Rodriguez F Oliver JL Marfn A Medina JR The general stochastic model of nucleotide substitution Journal of Theoretical Biology 1990 142 485 501 10.1016/S0022-5193(05)80104-3 2338834

[B33] Drummond AJ Rambaut A BEAST: Bayesian evolutionary analysis by sampling trees BMC Evolutionary Biology 2007 7 10.1186/1471-2148-7-214 17996036 PMC2247476

[B34] Suchard MA Weiss RE Sinsheimer JS Bayesian selection of continuous-time markov chain evolutionary models Molecular Biology and Evolution 2001 18 16 1001 1013 11371589 10.1093/oxfordjournals.molbev.a003872

[B35] Hasegawa M Kishino H Yano T Dating of human-ape splitting by a molecular clock of mitochondrial DNA Journal of Molecular Evolution 22 160 174 10.1007/BF02101694 3934395

[B36] Pond SLK Frost SDW A genetic algorithm approach to detecting lineage-specific variation in selection pressure Molecular Biology and Evolution 2005 22 3 478 485 10.1093/molbev/msi031 15509724

[B37] Pond SLK Posada D Gravenor MB Woelk CH Frost SDW Automated phylogenetic detection of recombination using a genetic algorithm Molecular Biology and Evolution 2006 23 10 1891 1901 10.1093/molbev/msl051 16818476

[B38] Kishino H Hasegawa M Evaluation of the maximum likelihood estimate of the evolutionary tree topologies from DNA sequence data, and the branching order in Hominodea Journal of Molecular Evolution 1989 29 170 179 10.1007/BF02100115 2509717

[B39] Martin DP Williamson C Posada D RDP2: recombination detection and analysis from sequence alignments Bioinformatics 2005 21 2 260 262 10.1093/bioinformatics/bth490 15377507

[B40] Martin D Rybicki E RDP: detection of recombination amongst aligned sequences Bioinformatics 2000 16 6 562 563 10.1093/bioinformatics/16.6.562 10980155

[B41] Padidam M Sawyer S Fauquet CM Possible emergence of new geminiviruses by frequent recombination Virology 1999 265 218 225 10.1006/viro.1999.0056 10600594

[B42] Salminen MO Carr JK Burke DS McCutchan FE Identification of breakpoints in intergenotypic recombinants of HIV type 1 by bootscanning AIDS Research and Human Retroviruses 1995 11 1423 1425 10.1089/aid.1995.11.1423 8573403

[B43] Maynard Smith J Analyzing the mosaic structure of genes Journal of Molecular Evolution 1992 34 126 129 1556748 10.1007/BF00182389

[B44] Posada D Crandall KA Evaluation of methods for detecting recombination from DNA sequences: computer simulations Proceedings of the National Academy of Sciences, USA 2001 98 13757 13762 10.1073/pnas.241370698 PMC61114 11717435

